# Palladium Supported on Porous Organic Polymer as Heterogeneous and Recyclable Catalyst for Cross Coupling Reaction

**DOI:** 10.3390/molecules27154777

**Published:** 2022-07-26

**Authors:** Guanying Shi, Zhenhua Dong

**Affiliations:** 1 Institute of Agricultural Products Processing, Henan Academy of Agricultural Sciences, Zhengzhou 450002, China; wendy1984@foxmail.com; 2 College of Chemistry and Chemical Engineering, Henan University of Technology, Lianhua Street 100, Zhengzhou 450001, China

**Keywords:** palladium, porous organic polymer, Heck reaction, nanoparticles, heterogeneous catalysis

## Abstract

Palladium immobilized on an amide and ether functionalized porous organic polymer (Pd@AEPOP) is reported to be an effective heterogeneous catalyst for the Heck cross-coupling reaction of aryl iodides with styrene for the synthesis of diphenylethene derivatives. Excellent yields can be obtained using a 0.8 mol% Pd catalyst loading under the optimized reaction condition. The heterogeneous Pd@AEPOP catalyst can also be applied on the Suzuki reaction and the reduction of nitroarene.

## 1. Introduction

As Nobel reactions, the Suzuki reaction and Heck reaction have a wide range of applications in organic synthetic chemistry [1,2,3,4]. The bi-aryl products are multipurpose building blocks in pharmaceuticals, agrochemicals and semiconductor materials such as organic light emitting diodes [5]. The cross-coupling reaction is one of the most important methods for the direct synthesis of related products [6]. In the past decades, a wide range of coupling reactions has been catalyzed effectively by palladium catalysts to form the carbon–carbon bond [7,8,9,10,11,12,13]. In order to reduce the reaction cost and expand the catalytic application, several metals have displayed analogous catalytic ability in this transformation [14]. Except for the noble metals, such as Au [15,16], Ru [17], Pt [18] and Ag [19], the low-cost transition metals, such as Cu [20], Ni [21], Co [22] and Fe [23], have also been investigated by chemists. However, compared with the Pd catalyst, other metal catalyzed coupling reactions still have many disadvantages, such as the low catalyst utilization rate, the ligands with complex structures, and the limited range of substrate scopes. The Pd-catalyzed coupling reactions are still the most popular research. However, palladium has a number of drawbacks, such as it being expensive, sensitive to air and moisture, and difficult to recover.

In order to improve the catalytic efficiency of Pd, remarkable research has been accomplished. Among this research, nano-catalysis is an emerging method [24,25,26,27,28]. Recently, considerable attention has been paid to the Pd nanoparticles (NPs). Several Pd nano-catalysts have displayed outstanding catalytic activity in cross-coupling reactions [29,30,31,32]. Existing studies indicated that the catalytic activity of Pd NPs is related to their intrinsic shape and size [33]. Hence, it is very important to confine the growth of NPs and control the size of NPs. However, due to the high surface energy, narrow sized metal particles tend to aggregate. It remains a standing challenge to obtain stable Pd NPs with a narrow size and thus outstanding catalytic activity. In our previous studies, we reported several porous organic polymers with diverse linked groups and well-defined pore structures [34,35,36]. In order to continue our efforts to extend the catalytic application of heterogeneous Pd NP catalysts, herein, we report a Pd NP supported on an amide and ether functionalized porous organic polymer (Pd@AEPOP) as an efficient and recyclable catalyst for Heck and Suzuki coupling.

## 2. Results and Discussion

**Scheme 1:**
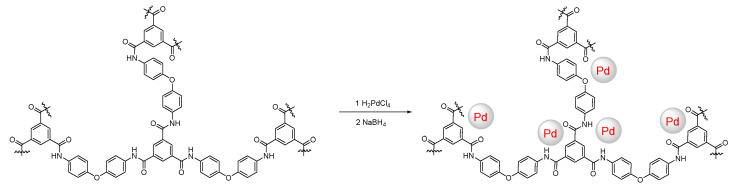
Preparation of Pd@AEPOP.

The synthetic method of Pd@AEPOP is shown in Scheme 1. The typical synthesis for AEPOP was described in the previous report [37]. Subsequently, the supported Pd nanoparticles’ (NPs) catalyst, named Pd@AEPOP, is prepared by mixing AEPOP with H2PdCl4 with reduction by NaBH4. The Pd content is 4.38% from ICP (inductively coupled plasma) analysis.

**Figure 1:**
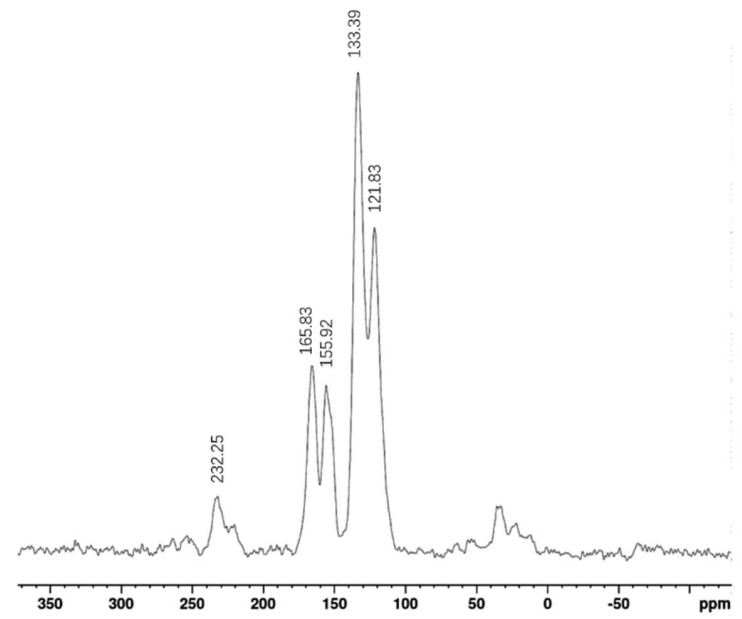
CP-MAS ^13^C NMR spectra of AEPOP.

Though the characterization of APOP has been well indicated. The molecular structure of AEPOP is also confirmed by the ^13^C CP-MAS solid state NMR spectrum. As shown in Figure 1, the characteristic resonance signals at 232.25, 165.83, 155.92, 133.39 and 121.83 ppm are observed. The signal at 232.25 corresponds to the carbonyl carbon, whereas the four strong signals that appeared from 165.83 to 121.83 ppm may originate from the four different aromatic carbons.

**Figure 2:**
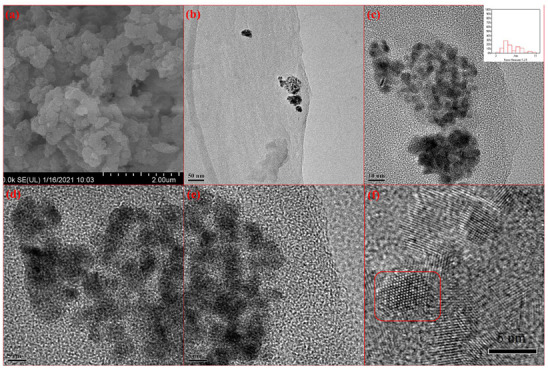
SEM and TEM characterizations of AEPOP and Pd@AEPOP. (**a**) SEM image of AEPOP, (**b**–**e**) Pd NPs in different scales, (**f**) diffraction fringe of Pd.

The porosity and morphology of AEPOP was described by SEM. As shown in Figure 2a, it is clear that AEPOP has a coralline-like morphology. The aperture in the surface can accommodate the Pd particles. The HR-TEM analysis shown in Figure 2b–f provides the morphology and shape of Pd@AEPOP. Figure 2b give the dispersity of Pd NPs at a large scale (50 nm). The dispersibility is unsatisfactory, and it looks like the Pd particles agglomerate. However, the enlarged photo, Figure 2c, shows that the Pd particles are separated from each other. According to the statistics regarding the Pd particles, an average particle size of approximately 5–8 nm is observed. Figure 2d,e show the clear morphology and the shape of Pd NPs with enlarged images (5 nm). It is clear that the Pd particles are nanoparticles. The diffraction fringe of Pd is clearly visible from Figure 2f in the red pane. The boundary is distinct between the two different Pd particles.

**Figure 3:**
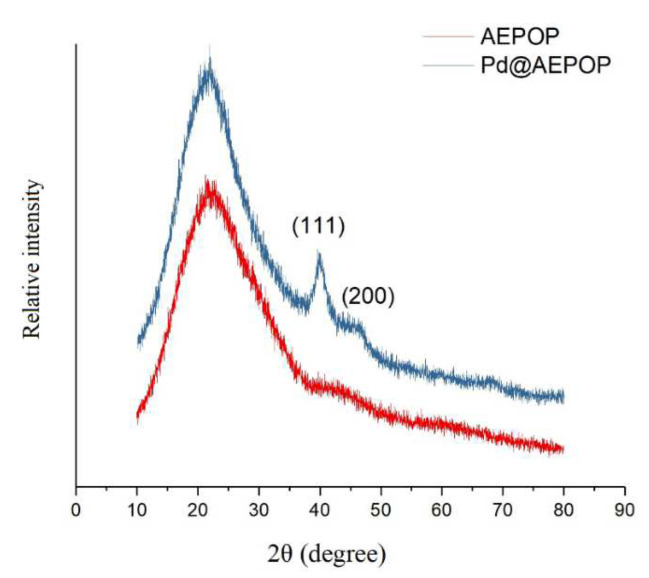
PXRD patterns of AEPOP and Pd@AEPOP.

Then the structural regularity and crystalline nature of Pd@AEPOP was determined by powder PXRD analysis. The PXRD pattern of Pd@AEPOP (blue line) gives new small peaks at 2θ values 39.84 and 45.88 as shown in Figure 3. The values can be assigned to the refractions of (111) and (200) of Pd NPs, respectively.

The Pd@AEPOP was also confirmed by FTIR spectroscopy. As shown in Figure S1 in the Supplementary Materials, the IR spectroscopy of Pd@AEPOP (blue line) has no obvious change compared with AEPOP (red line). However, the Pd content is higher than that in our previous work. The results indicate that the Pd NPs may be embedded into the porous material by physical absorption.

In order to evaluate the catalytic activity of the Pd@AEPOP catalyst for cross-coupling reactions, the Heck reaction of 4-methyl iodobenzene with styrene is selected as the model reaction. Several conditions are examined for the Heck reaction, and the results are summarized in Table 1. Using 20 mg of the Pd@AEPOP catalyst, the screening of the solvents reveals that DMF is the best solvent with a 40% yield (entries 1–4). Then various bases were tested (entries 5, 6). The organic base Et_3_N is found to be the best base with a 55% yield. Then a 99% yield can be achieved by the screening of temperature, at 120 °C (entry 9). The best yield of 95% is obtained by prolonging the reaction time to 10 h (entry 10).

With the optimized reaction conditions in hand, various substitutional aryl iodides were tested and the results are summarized in Table 2. Using styrene as a standard substance, an excellent yield can be achieved for different aryl iodides containing both electron-donating (Me, *t*Bu) and electron-withdrawing (Cl) groups (Table 2, entries 1–4). Then the ethylacrylate is also used as a standard substance, and various substitutional aryl iodides also give excellent yields (Table 2, entries 5–11). The aryl bromide and chloride substrates were also extended. Unfortunately, no products were observed.

**Scheme 2:**
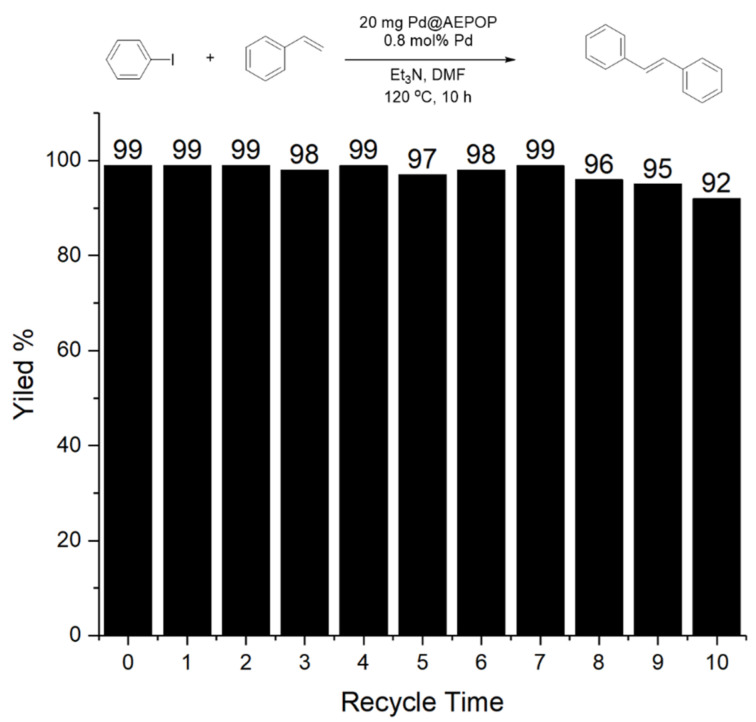
The recyclability of Pd@AEPOP.

Then the recyclability of the Pd@AEPOP catalyst is tested. After the end of the model reaction, the Pd@AEPOP was separated by simple centrifugation. Then the catalyst was used for the next catalysis after washing by DMF and drying. As shown in Scheme 2, the catalyst can be recycled nine times. The decrease in activity may result from the loss of the Pd in the recycling processes. Hence, we also carried out the ICP analysis for the final Pd@AEPOP catalyst after the tenth use and the Pd content was 3.96%. In view of the possible physical adsorption of Pd particles on the porous organic polymer, the results of recyclability are satisfactory.

**Scheme 3:**
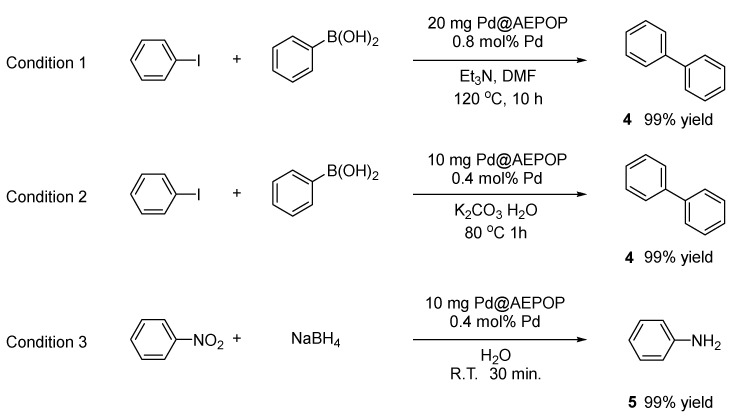
Catalytic application extension of Pd@AEPOP.

Most existing reports about Pd NPs’ catalysis focus mainly on the Suzuki cross-coupling reaction. Hence, the performance of the Pd@AEPOP catalyst for the Suzuki reaction is investigated, and the results are shown in Scheme 3. Under the standard condition in this paper, a 99% yield is obtained (condition 1). The Suzuki reaction also proceeds under more the mild and eco-friendly condition 2 using water as a solvent. The Pd@AEPOP catalyst can also be applied for the reduction of nitroarenes using NaBH_4_ as a reductant with 99% yield.

## 3. Experimental Section

### 3.1. Materials

The amide and ether functionalized porous organic polymer was prepared via the reaction of 4,4′-diaminodiphenyl ether with 1,3,5-benzenetricarbonyl chloride. The AEPOP was well characterized in our previous report.

### 3.2. Synthesis of Pd@AEPOP

In total, 10 mL of H_2_PdCl_4_ (12 mg Pd/mL) was added to the mixture of 2 g AEPOP with 300 mL H_2_O under stirring at room temperature. The reaction was stirred for 24 h. Then a solution of 856 mg NaBH_4_ in 100 mL H_2_O was added to the reaction slowly. The resulted mixture was stirred for 12 h at room temperature. Finally, the solid was filtered and washed by MeOH and water three times. The product was dried under vacuum at 130 °C for further use.

### 3.3. General Procedure for Heck Reaction

Solutions of 20 mg Pd@AEPOP, 1 mmol iodobenzene, 1.2 mmol styrene, 1.5 mmol Et_3_N were mixed with 2 mL DMF in glass tube. Then the reaction was carried by 120 °C for 10 h. After reaction, the mixture was cooled and extracted with 20 mL diethyl ether three times. The combined organic layer was dried (Na_2_SO_4_) and concentrated to give crude product. The desired product was purified by flash column chromatography on silica gel (petroleum ether and ethyl acetate).

### 3.4. General Procedure for Recycling Experiment

Solutions of 20 mg Pd@AEPOP, 1 mmol iodobenzene, 1.2 mmol styrene, 1.5 mmol Et_3_N were mixed with 2 mL DMF in glass tube (Scheme 2). Then the reaction was carried out at 12 °C for 10 h. After the reaction, the solid was separated by centrifugation. The liquid was further processed according to Section 3.3 to obtain the yield. The solid was washed by DMF and dried. Then the reclaimed catalyst was reused next time.

### 3.5. General Procedure for Suzuki Reaction 

Condition 1: 20 mg Pd@AEPOP, 1 mmol iodobenzene, 1.5 mmol arylboronic acids, and 1.5 mmol Et_3_N were mixed in 2.0 mL DMF in glass tube (Scheme 3). Then the reaction was carried out at 120 °C for 10 h. After reaction, the mixture was cooled and extracted with 20 mL diethyl ether three times. The combined organic layer was dried (Na_2_SO_4_) and concentrated to give crude product. The desired product was purified by flash column chromatography on silica gel (petroleum ether).

Condition 2: 10 mg Pd@AEPOP, 1 mmol iodobenzene, 1.5 mmol arylboronic acids, and 2 mmol K_2_CO_3_ were mixed in 2.0 mL water in glass tube (Scheme 3). Then the reaction was carried out at 80 °C for 1 h. After reaction, the mixture was cooled and extracted with 20 mL diethyl ether three times. The combined organic layer was dried (Na_2_SO_4_) and concentrated to give crude product. The desired product was purified by flash column chromatography on silica gel (petroleum ether).

### 3.6. General Procedure for Reduction of Nitroarene

Solutions of 10 mg Pd@AEPOP, 1 mmol nitroarenes were mixed with 2.0 mL water. Then 5 mmol NaBH_4_ was added to the reaction slowly (Scheme 3). Then the reaction was stirred 30 min at room temperature. The reaction was extracted with 20 mL diethyl ether three times. The combined organic layer was dried (Na_2_SO_4_) and concentrated to give crude product. The desired product was purified by flash column chromatography on silica gel (petroleum ether and ethyl acetate).

## 4. Conclusions

In conclusion, an efficient method for the Heck cross-coupling reaction of aryl iodides with styrene for the synthesis of a di-aryl ethene derivative using Pd@AEPOP as an active and reusable catalyst has been developed. Under optimized reaction conditions, an excellent yield was achieved by a 0.8 mol% palladium catalyst loading. The Pd catalyst can be recycled nine times without an obvious decrease in activity. Moreover, the catalyst can also be applied for the Suzuki reaction and reduction of nitroarene.

## Figures and Tables

**Table 1:** Identification of reaction conditions ^a^.

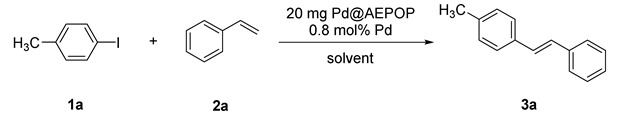					
Entry	Base	Solvent	Temp.	Time (h)	Yield ^b^
1	K_2_CO_3_	DMSO	90	5	Trace
2	K_2_CO_3_	DMF	90	5	36
3	K_2_CO_3_	H_2_O	90	5	10
4	K_2_CO_3_	DMF	90	5	40
5	Et_3_N	DMF	90	5	55
6	Cs_2_CO_3_	DMF	90	5	Trace
7	Et_3_N	DMF	100	5	63
8	Et_3_N	DMF	110	5	71
9	Et_3_N	DMF	120	5	75
10	Et_3_N	DMF	120	10	95

^a^ Reaction conditions: 4-methyl iodobenzene 1 mmol, styrene 1.2 mmol, Pd@AEPOP (20 mg, 0.8 mol% Pd content), base 1.5 mmol, solvent 2 mL. ^b^ Isolated yield.

**Table 2:** Substrate scopes of Heck reactions ^a^.

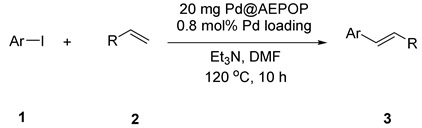			
Entry	ArI	R	Yield ^b^
1	4-Me-Ph	Ph	95
2	Ph	Ph	99
3	4-*t*Bu-Ph	Ph	93
4	3-Cl-Ph	Ph	98
5	Ph	COOEt	98
6	3-Cl-Ph	COOEt	99
7	4-Cl-Ph	COOEt	99
8	2-Me-Ph	COOEt	92
9	3-Me-Ph	COOEt	98
10	4-Me-Ph	COOEt	99
11	4-MeO-Ph	COOEt	99

^a^ Aryl iodides (1 mmol), alkenes (1.2 mmol, 1.2 equiv.), Et_3_N (1.5 mmol, 1.5 equiv.), DMF (2 mL), Pd@AEPOP (20 mg, 0.8 mol% Pd content), 120 °C, 10 h. ^b^ Isolated yield.

## Data Availability

The data presented in this study are available in insert article or Supplementary Materials here.

## Supplementary Materials

The following are available online at https://www.mdpi.com/article/10.3390/molecules27154777/s1, Figure S1: FT-IR spectra of AEPOP and Pd@AEPOP.
