# Supplementary material for: Palladium Supported on Porous Organic Polymer as Heterogeneous and Recyclable Catalyst for Cross Coupling Reaction
Source: Molecules. 2022 Jul 26;27(15):4777. doi: 10.3390/molecules27154777 (PMC9332046; doi:10.3390/molecules27154777)

molecules

Supplementary Materials

Palladium Supported on Porous Organic Polymer as Heterogeneous and Recyclable Catalyst for Cross Coupling Reaction

Guanying Shi [1] and Zhenhua Dong [2,*]

[1] Institute of Agricultural Products Processing, Henan Academy of Agricultural Sciences, Zhengzhou 450002, China; wendy1984@foxmail.com
[2] College of Chemistry and Chemical Engineering, Henan University of Technology, Lianhua Street 100, Zhengzhou 450001, China
* Correspondence: zhdong@haut.edu.cn

1. General information

All reagents were of analytical grade and purchased from Innochem Industrial Inc. The chemicals were used without further purification. NMR spectra were recorded at room temperature in $CDCl_3$ or DMSO-d6 on 400 MHz spectrometers.

The IR spectra were obtained with KBr pellets in the range of 400-4000 cm^{-1} using a PerkinElmer Frontier (Spectrum TWO) spectrometer. The crystallographic structure was characterized by X-ray diffraction (XRD) on an X-ray powder diffractometer (Rigaku SmartLab9, Japan) using Cu Ka radiation ($\lambda = 1.5406$ Å). The morphology of nanopowders was characterized by using a scanning electron microscopy (SEM, Zeiss Sigma 300, Smart EDX). The Brunauer-Emmett-Teller (BET) specific surface area and porosity analysis were carried out using Micromeritics (ASAP 2460) at 77 K with nitrogen. TEM images were recorded on FEI Tecnai G2 F2. Inductively Coupled Plasma Atomic Emission Spectroscopy (ICP-AES) was tested on Agilent 5110. Thermo gravimetric analyses (TGA) were performed on a thermogravimetric/differential thermal analyzer (NETZSCH TG209F1) by heating the samples at 10 °C min^{-1} to 800 °C under nitrogen atmosphere.

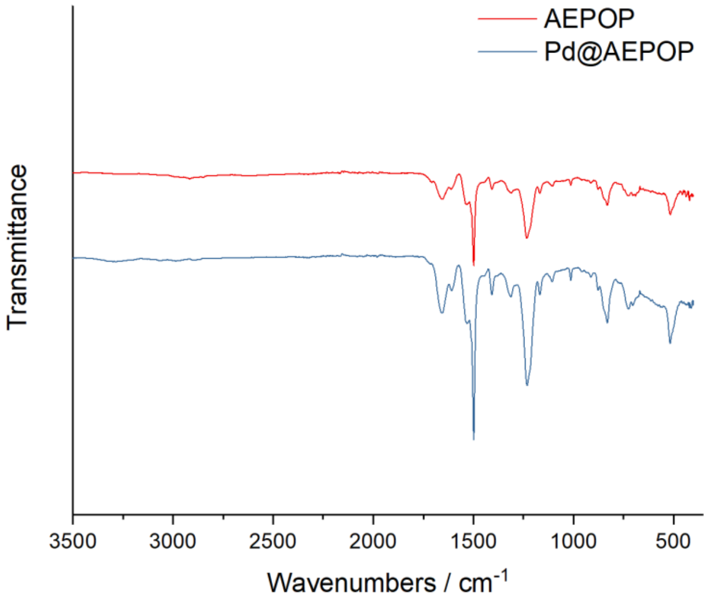

Figure S1. FT-IR spectra of AEPOP and Pd@AEPOP.

2 Spectra data of products

3a: ^{1}H NMR (600 MHz, Chloroform-*d*) δ 7.54 (d, *J* = 7.7 Hz, 2H), 7.45 (d, *J* = 7.8 Hz, 2H), 7.39 (t, *J* = 7.6 Hz, 2H), 7.33 – 7.25 (m, 1H), 7.21 (d, *J* = 7.7 Hz, 2H), 7.17 – 7.06 (m, 2H), 2.40 (s, 3H).

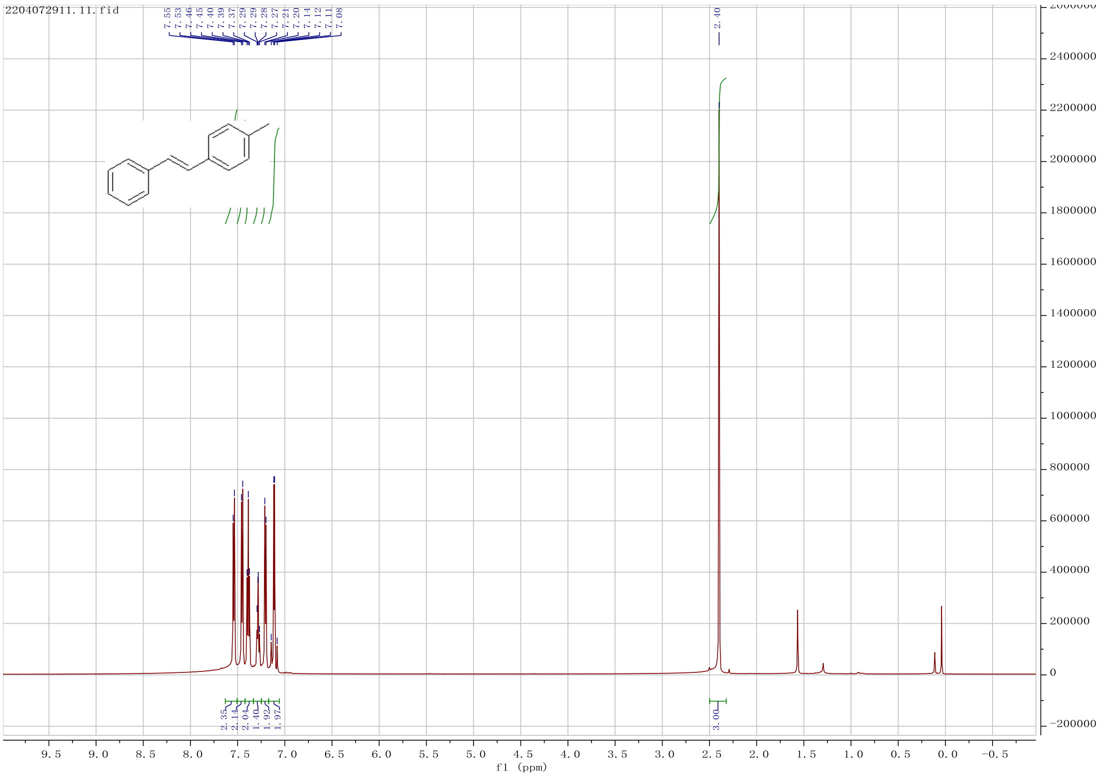

3b: ^{1}H NMR (400 MHz, Chloroform-d) δ 7.65 – 7.53 (m, 2H), 7.41 (t, J = 7.6 Hz, 2H), 7.36 – 7.27 (m, 1H), 7.16 (s, 1H).

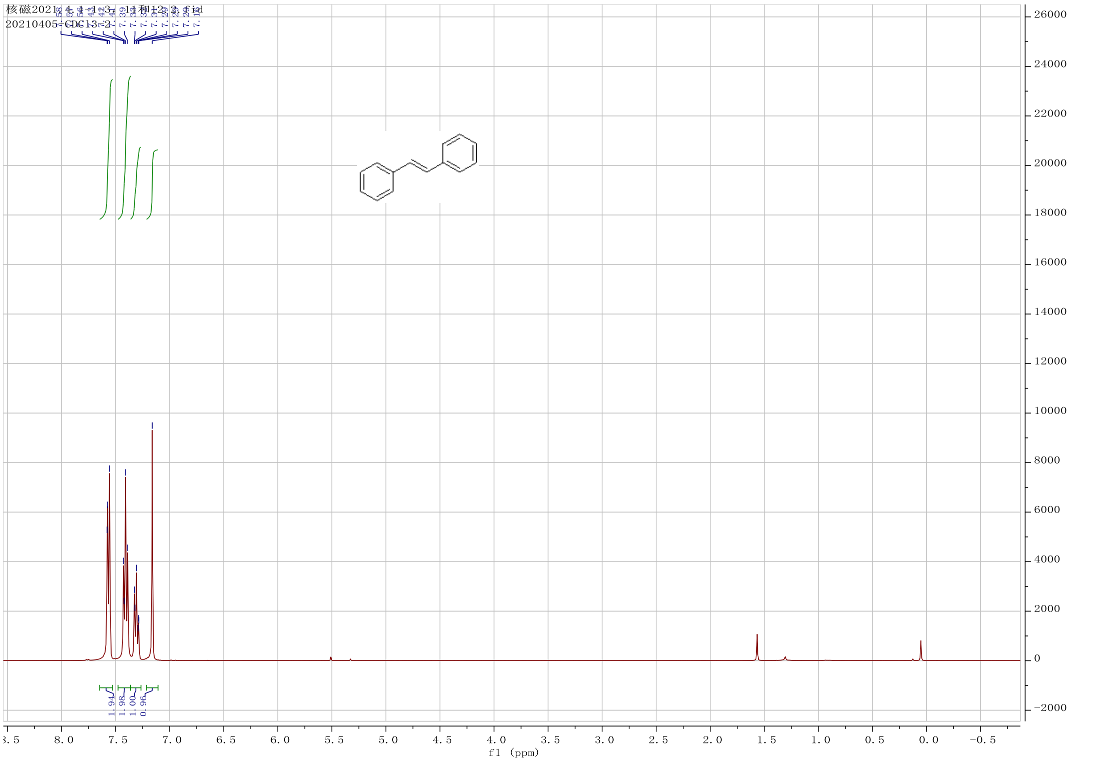

3c:^{1}H NMR (400 MHz, Chloroform-*d*) δ 7.60 – 7.53 (m, 2H), 7.53 – 7.48 (m, 2H), 7.48 – 7.36 (m, 4H), 7.36 – 7.26 (m, 1H), 7.14 (d, *J* = 2.7 Hz, 2H), 1.38 (s, 9H).

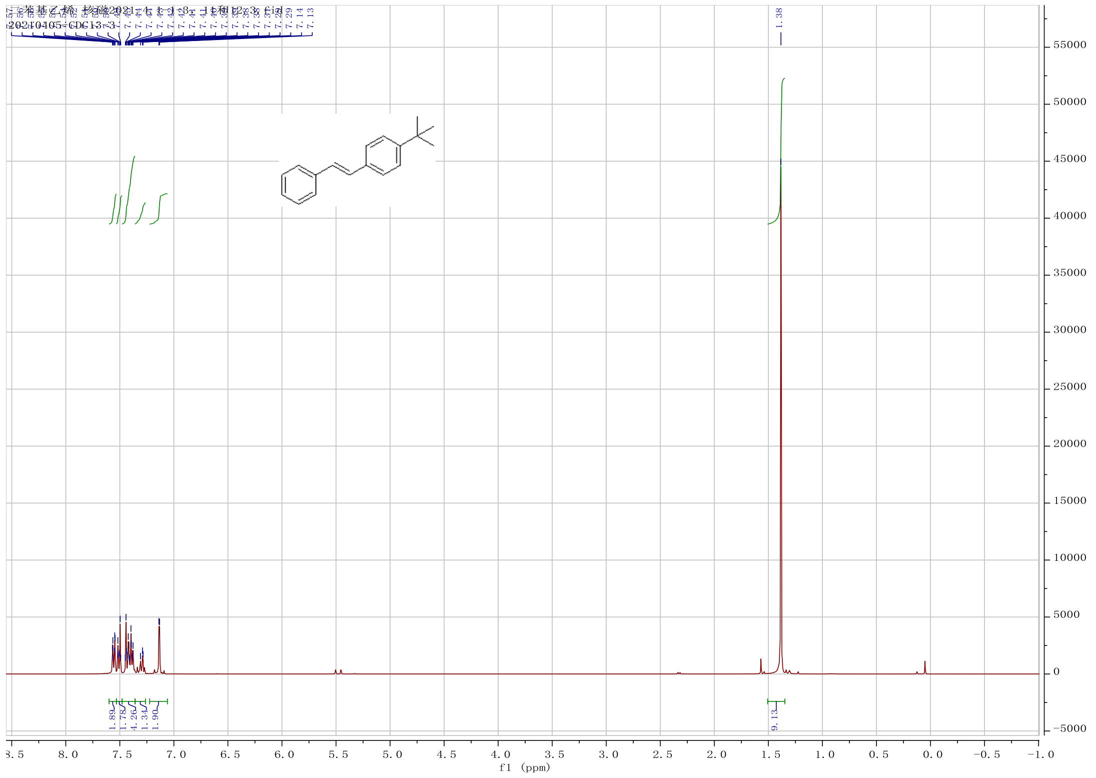

3d:^{1}H NMR (400 MHz, Chloroform-*d*) δ 7.55 (dd, *J* = 6.9, 1.8 Hz, 3H), 7.46 – 7.37 (m, 4H), 7.32 (td, *J* = 7.4, 3.5 Hz, 2H), 7.29 – 7.24 (m, 1H), 7.11 (q, *J* = 16.3 Hz, 2H).

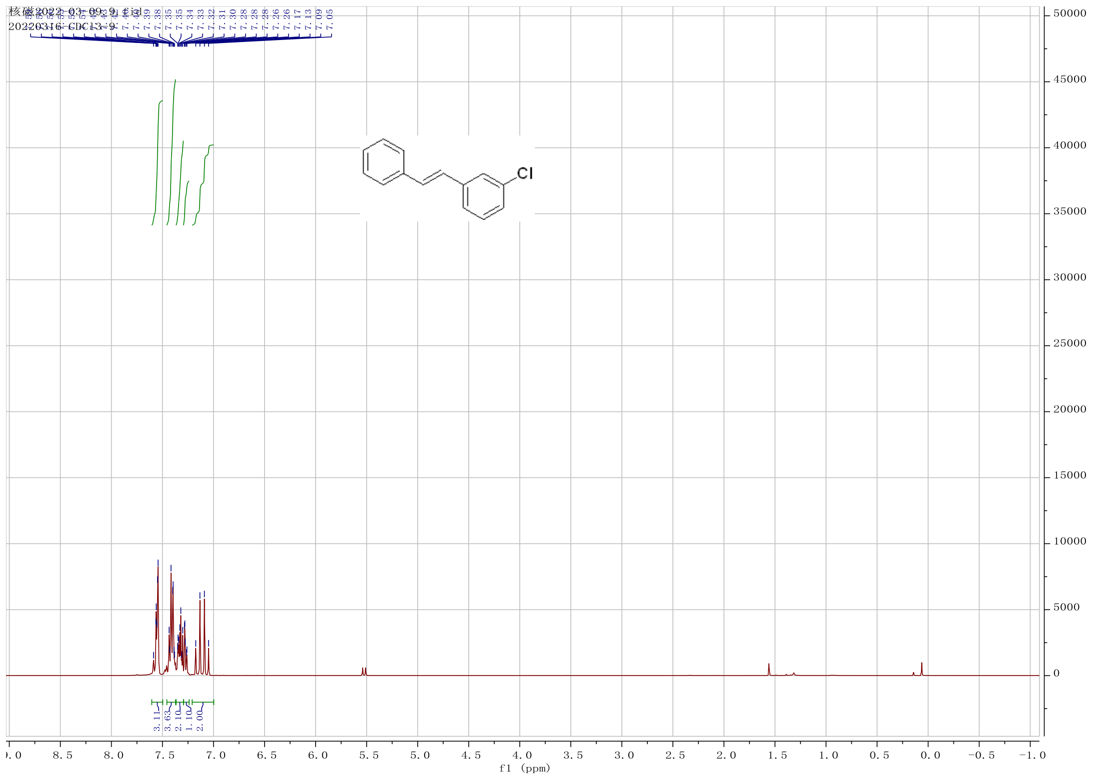

3e: ^{1}H NMR (400 MHz, Chloroform-d) δ 7.71 (d, J = 16.0 Hz, 1H), 7.62 – 7.50 (m, 2H), 7.48 – 7.35 (m, 3H), 6.46 (d, J = 16.0 Hz, 1H), 4.29 (q, J = 7.1 Hz, 2H), 1.36 (t, J = 7.1 Hz, 3H).

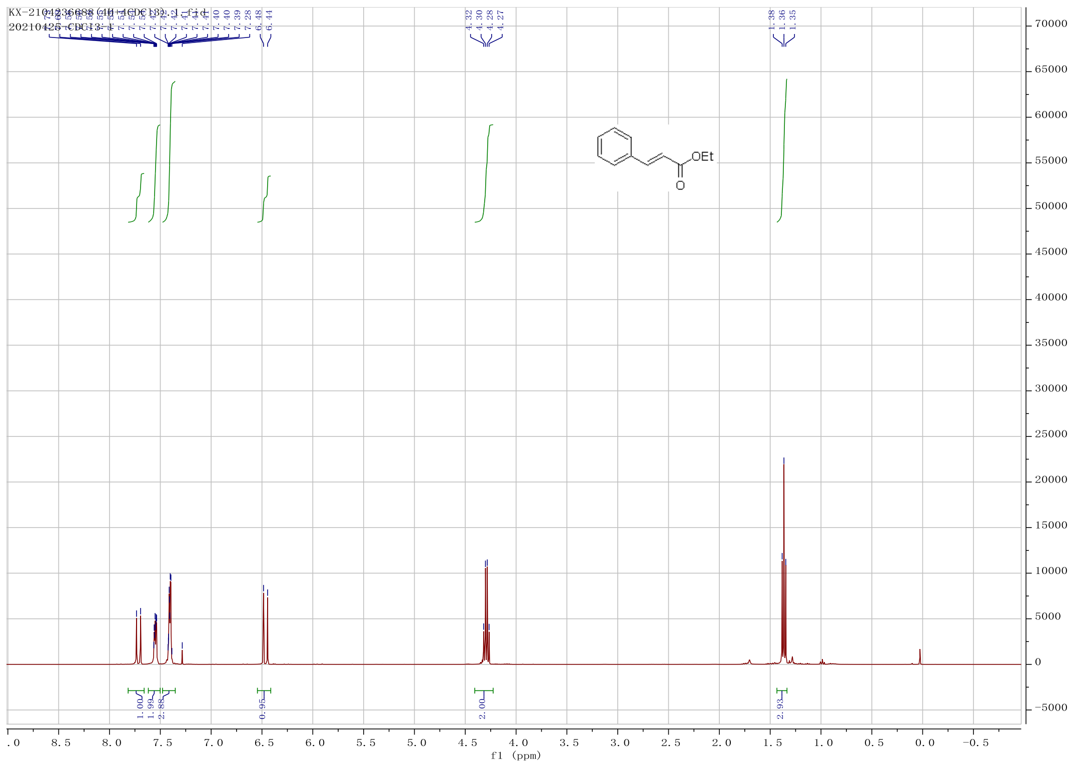

3f: ^{1}H NMR (400 MHz, Chloroform-d) δ 7.64 (d, J = 16.0 Hz, 1H), 7.53 (t, J = 1.9 Hz, 1H), 7.45 – 7.31 (m, 3H), 6.46 (d, J = 16.0 Hz, 1H), 4.29 (q, J = 7.1 Hz, 2H), 1.36 (t, J = 7.1 Hz, 3H).

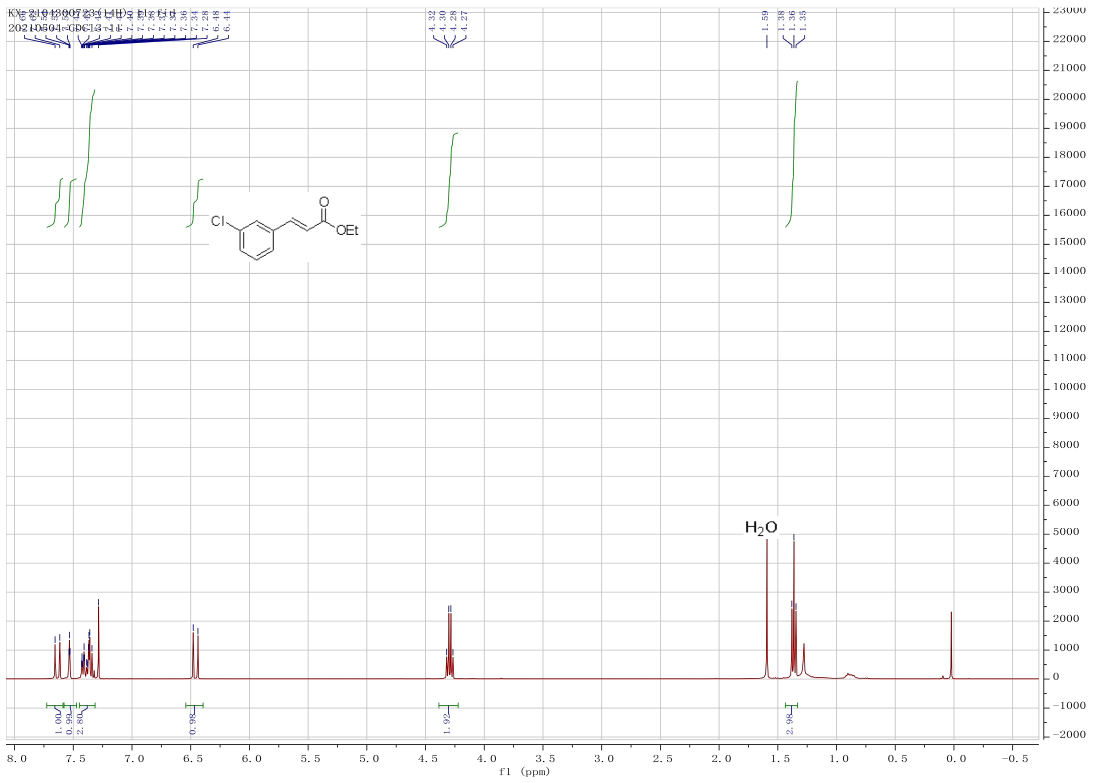

3g: ^{1}H NMR (400 MHz, Chloroform-d) δ 7.65 (d, J = 16.0 Hz, 1H), 7.55 – 7.45 (m, 2H), 7.45 – 7.37 (m, 2H), 6.43 (d, J = 16.0 Hz, 1H), 4.29 (q, J = 7.1 Hz, 2H), 1.36 (t, J = 7.1 Hz, 3H).

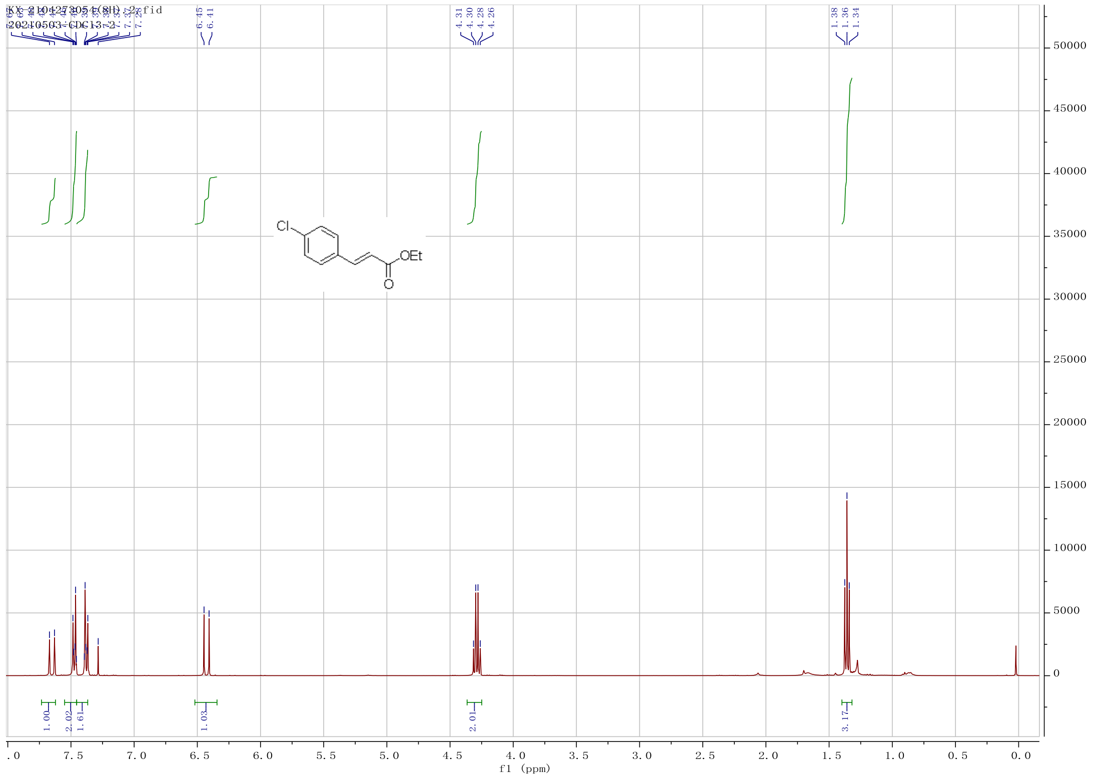

3h: ^{1}H NMR (400 MHz, Chloroform-d) δ 8.00 (d, J = 15.9 Hz, 1H), 7.64 – 7.53 (m, 1H), 7.35 – 7.18 (m, 4H), 6.38 (d, J = 15.9 Hz, 1H), 4.30 (q, J = 7.1 Hz, 2H), 2.47 (s, 3H), 1.37 (t, J = 7.1 Hz, 3H).

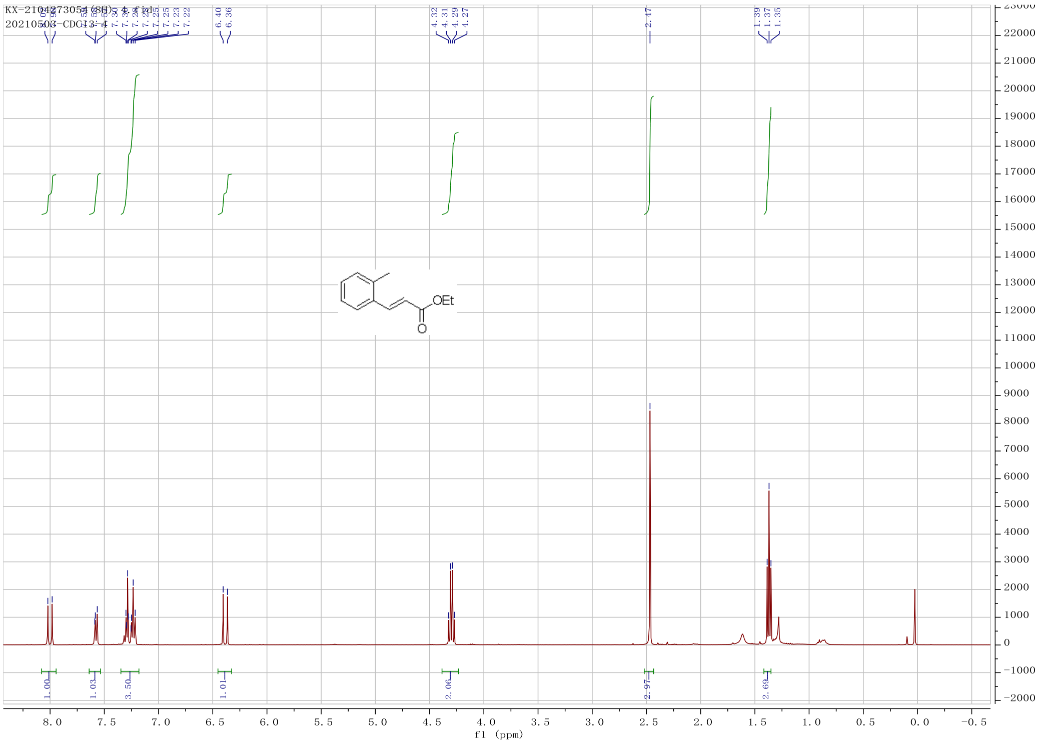

3i: ^{1}H NMR (400 MHz, Chloroform-d) δ 7.68 (d, J = 16.0 Hz, 1H), 7.36 (d, J = 6.9 Hz, 2H), 7.33 – 7.25 (m, 1H), 7.22 (d, J = 7.5 Hz, 1H), 6.45 (d, J = 16.0 Hz, 1H), 4.29 (q, J = 7.1 Hz, 2H), 2.39 (s, 3H), 1.36 (t, J = 7.1 Hz, 3H).

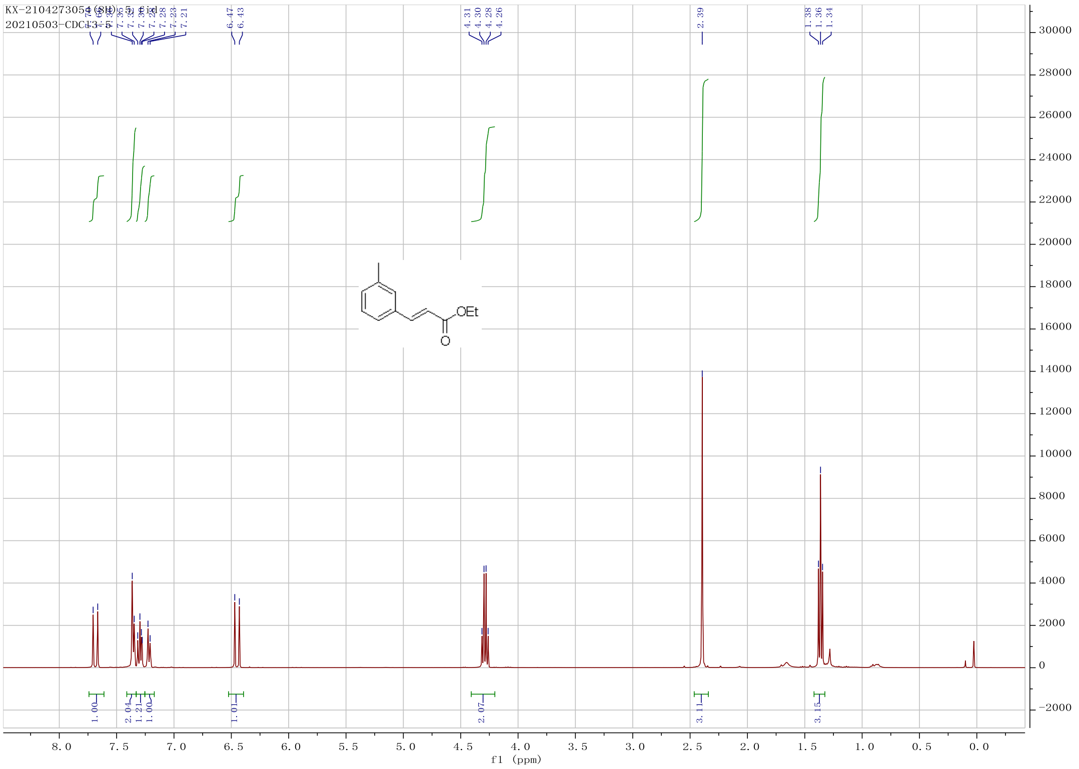

3j: ^{1}H NMR (400 MHz, Chloroform-d) δ 7.69 (d, J = 16.0 Hz, 1H), 7.54 – 7.40 (m, 2H), 7.21 (d, J = 7.9 Hz, 2H), 6.42 (d, J = 16.0 Hz, 1H), 4.28 (q, J = 7.1 Hz, 2H), 2.39 (s, 3H), 1.36 (t, J = 7.1 Hz, 3H).

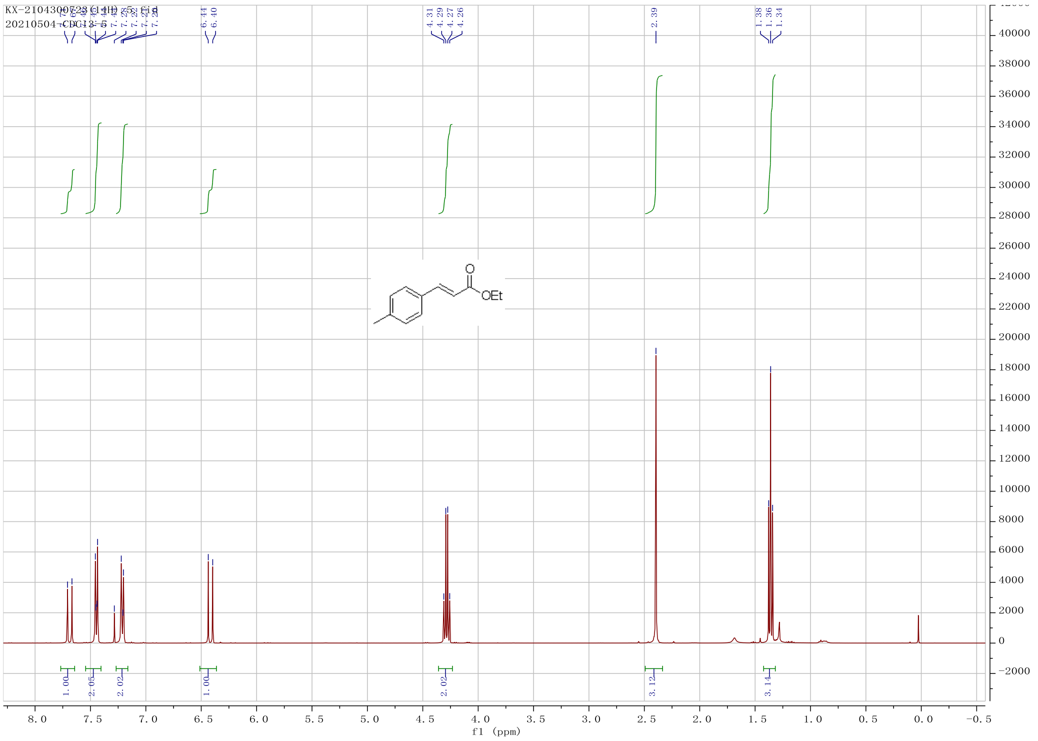

3k: ^{1}H NMR (400 MHz, Chloroform-d) δ 7.66 (d, J = 16.0 Hz, 1H), 7.56 – 7.45 (m, 2H), 7.01 – 6.83 (m, 2H), 6.33 (d, J = 15.9 Hz, 1H), 4.27 (q, J = 7.1 Hz, 2H), 3.85 (s, 3H), 1.35 (t, J = 7.1 Hz, 3H).

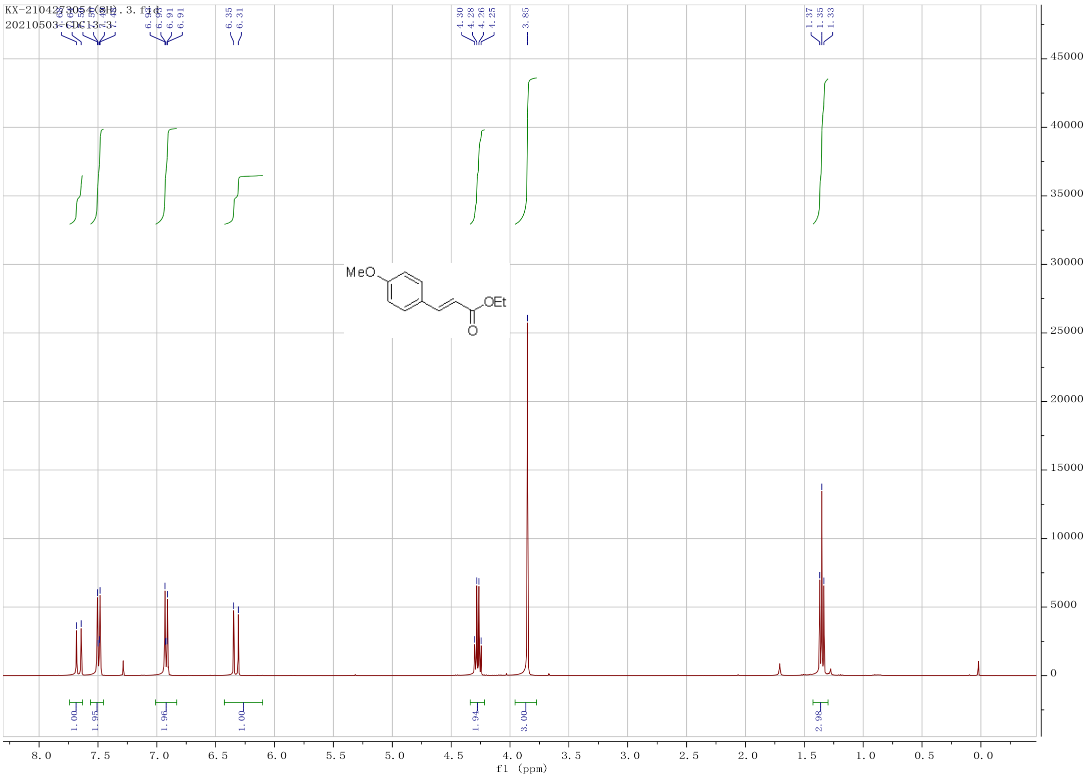

4:^{1}H NMR (400 MHz, Chloroform-d) δ 7.70 – 7.62 (m, 2H), 7.55 – 7.45 (m, 2H), 7.45 – 7.37 (m, 1H).

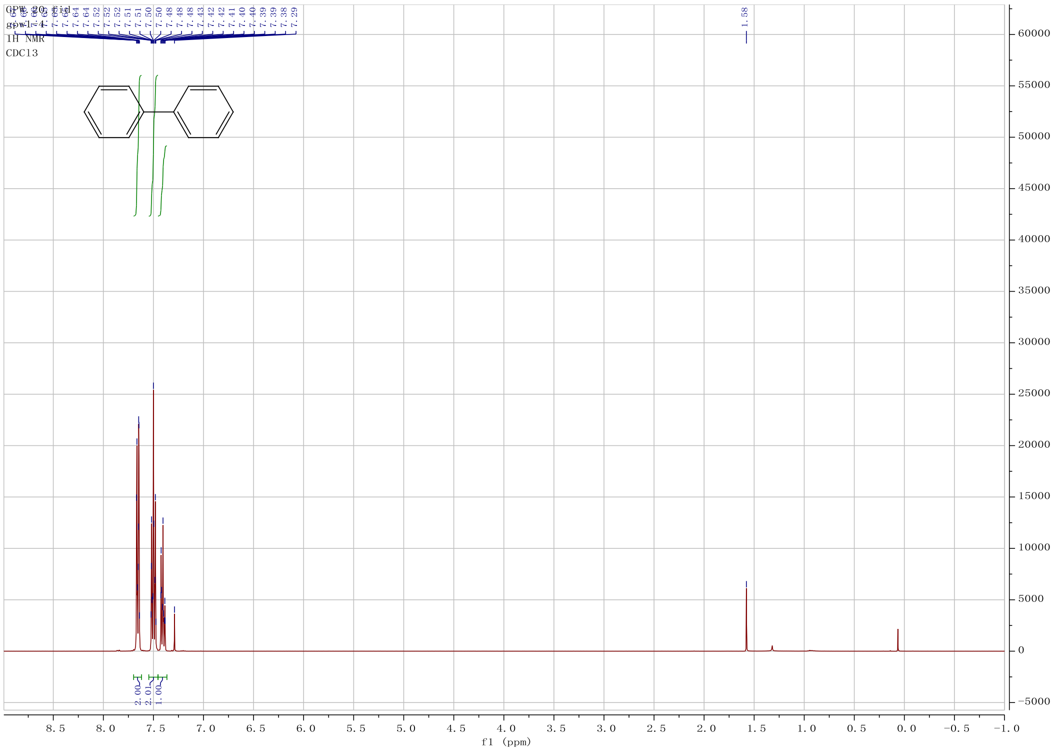

5:^{1}H NMR (600 MHz, Chloroform-d) δ 7.44 – 7.31 (m, 2H), 6.96 (tt, J = 7.3, 1.1 Hz, 1H), 6.87 – 6.77 (m, 2H), 3.74 (s, 2H).

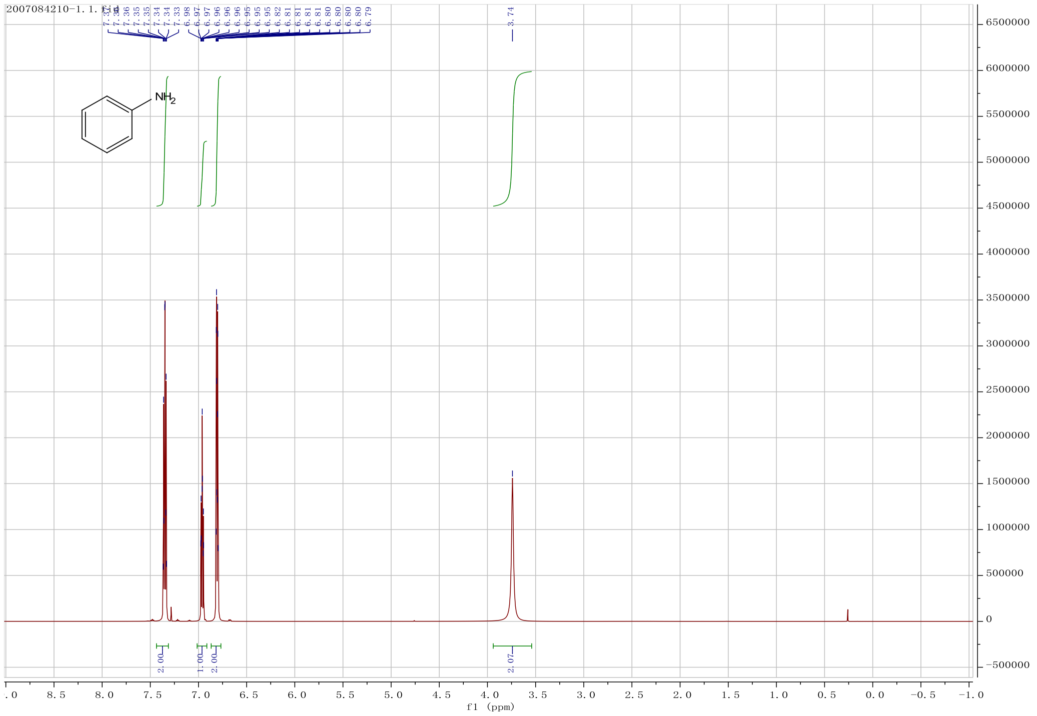

Supplement: Supplementary file 1 [file molecules-27-04777-s001.zip › molecules-1828337-supplementary.pdf]
